# A *Drosophila* model to investigate the neurotoxic side effects of radiation exposure

**DOI:** 10.1242/dmm.019786

**Published:** 2015-07-01

**Authors:** Lisa J. Sudmeier, Steven P. Howard, Barry Ganetzky

**Affiliations:** 1 Laboratory of Genetics, University of Wisconsin-Madison, 425 Henry Mall, Madison, WI 53706, USA; 2 Department of Human Oncology, University of Wisconsin School of Medicine and Public Health, 600 Highland Ave., Madison, WI 53792, USA

**Keywords:** Gene association studies, Radiosensitivity, Cell death, Neuropathology

## Abstract

Children undergoing cranial radiation therapy (CRT) for pediatric central nervous system malignancies are at increased risk for neurological deficits later in life. We have developed a model of neurotoxic damage in adult *Drosophila* following irradiation during the juvenile stages with the goal of elucidating underlying neuropathological mechanisms and of ultimately identifying potential therapeutic targets. Wild-type third-instar larvae were irradiated with single doses of γ-radiation, and the percentage that survived to adulthood was determined. Motor function of surviving adults was examined with a climbing assay, and longevity was assessed by measuring lifespan. Neuronal cell death was assayed by using immunohistochemistry in adult brains. We also tested the sensitivity at different developmental stages by irradiating larvae at various time points. Irradiating late third-instar larvae at a dose of 20 Gy or higher impaired the motor activity of surviving adults. A dose of 40 Gy or higher resulted in a precipitous reduction in the percentage of larvae that survive to adulthood. A dose-dependent decrease in adult longevity was paralleled by a dose-dependent increase in activated Death caspase-1 (Dcp1) in adult brains. Survival to adulthood and adult lifespan were more severely impaired with decreasing larval age at the time of irradiation. Our initial survey of the *Drosophila* Genetic Reference Panel demonstrated that differences in genotype can confer phenotypic differences in radio-sensitivity for developmental survival and motor function. This work demonstrates the usefulness of *Drosophila* to model the toxic effects of radiation during development, and has the potential to unravel underlying mechanisms and to facilitate the discovery of novel therapeutic interventions.

## Abstract (precis)

**Highlighted Article:** To model delayed neurological deficits resulting from pediatric cranial radiation therapy, neurotoxic damage in adult *Drosophila* is assessed following larval irradiation with the goal of elucidating underlying pathological mechanisms.

## INTRODUCTION

Cranial radiation therapy (CRT) is a mainstay of treatment for brain tumors, along with surgery and chemotherapy. CRT preferentially kills malignant cells by inducing DNA damage that results in cell-cycle arrest and death of dividing cells. Therapy is often designed to target the tumor resection bed and a margin; lower doses of radiation are sometimes delivered to the rest of the brain to target micrometastases. Prophylactic CRT might also be administered to high-risk individuals to reduce or prolong the time to development of brain metastases. CRT has consistently prolonged survival in children with brain tumors, and as therapies have improved, the rate of cure and the length of disease-free survival has also increased, especially in children with brain tumors (Armstrong, 2010). As therapy has become more effective and affected individuals live longer, the long-term sequelae of this treatment, particularly in children, have received increasing attention. Individuals who undergo CRT before the age of 18 have a particularly increased incidence of many neurological and cognitive side effects, including impairments of memory, attention, visuo-spatial processing, learning ability, and motor control and dexterity, in addition to increased prevalence of seizure disorders (Armstrong et al., 2009; Edelstein et al., 2011b; Ellenberg et al., 2009; Mulhern et al., 2005; Packer et al., 2003; Redmond et al., 2013). Pathological analysis has demonstrated CRT-associated changes in the brain during development, including disrupted neurogenesis, demyelination, cortical atrophy and damage to the microvasculature (Ball et al., 1992; Jain et al., 2005; Monje et al., 2002; Schuitema et al., 2013). As increasingly effective therapy results in more pediatric brain tumor survivors, there is an increasing need for radioprotective strategies to reduce the damage to healthy tissue during treatment.

For decades, thiol compounds have been studied for their radioprotective properties, which are mediated through DNA binding and scavenging of reactive oxygen species (Hosseinimehr, 2007). These compounds effectively confer radioprotection in preclinical animal models but have limited clinical usefulness because they are difficult to administer and have a number of toxic side effects (Hosseinimehr, 2007; McAleer et al., 2005; Srinivasan et al., 2002). Thus, there is considerable interest in developing related but less toxic compounds that still provide radioprotection.

Identification of novel therapeutic targets offers another approach for developing improved radioprotective agents with reduced toxicity. These targets can differ among tissues and might offer a more specific and effective strategy for protecting healthy cells from high doses of therapeutic radiation. In order to identify potential targets for drug development, it is necessary to have a more complete and detailed understanding of the key biochemical pathways and molecular mechanisms that underlie the immediate and long-term neurotoxic effects of radiation exposure. Unbiased forward genetic screens in *Drosophila* have been crucial to the molecular dissection of other complex biological mechanisms, including autophagy, programmed cell death, the cell cycle and embryonic patterning (Mulakkal et al., 2014; Jenkins et al., 2013; Hérranz and Milán, 2008; Nüsslein-Volhard and Wieschaus, 1980). Using an appropriate experimental model, this approach should be equally valuable in deciphering the mechanism(s) of the acute and long-term sequelae of radiation during development. Previous radiation studies in *Drosophila* have identified lethal doses for larvae and have examined the effects of irradiating adults (Balock et al., 1963; Ogaki and Nakashima-Tanaka, 1966; Parsons et al., 1969; Pyo et al., 2014; Vaisnav et al., 2014; Westerman and Parsons, 1973), but the long-term effects on adults of radiation exposure during larval development have not been examined. Here, we show that many of the important neurotoxic side effects resulting from radiation therapy during development in humans can be reasonably mimicked in *Drosophila*. Moreover, we demonstrate that genetic background substantially affects radiosensitivity. These results demonstrate that *Drosophila* can serve as a useful experimental model for studying the neurotoxic consequence of radiation exposure, and ultimately for identifying specific genes and proteins involved in the molecular mechanism of radiation-induced damage.

**TRANSLATIONAL IMPACT**

**Clinical issue**

Children who undergo cranial radiation therapy (CRT) to treat central nervous system (CNS) malignancies are at increased risk for neurocognitive deficits, impaired coordination and motor control, and seizure disorders. The severity of these side effects often worsens as the age of treatment decreases. For decades, thiol compounds have been studied for their radioprotective properties, but they have limited clinical usefulness because they are difficult to administer and have a number of toxic side effects. Thus, there is considerable interest in trying to develop new radioprotective agents. One approach is to characterize the molecular pathways underlying radiation-induced neurotoxicity in order to identify novel therapeutic targets for enhancing radioprotection in healthy tissue. *Drosophila* is an ideal model for the molecular dissection of such complex biological mechanisms.

**Results**

In this work, the authors investigate the neurotoxic effects of radiation exposure in *Drosophila* at different developmental stages and on different genetic backgrounds. Irradiating *Drosophila* during the larval stage produces measurable phenotypes in adult flies that parallel those seen in survivors of CNS malignancies who received CRT during childhood. Irradiating late third-instar larvae at a dose of 40 gray (Gy, the unit in which the absorption of ionizing radiation is measured) or higher results in a sharp reduction in the percentage of larvae that survive and develop into adults. A dose of 20 Gy or higher impairs motor activity of surviving adults. A dose-dependent decrease in adult longevity is paralleled by a dose-dependent increase in activated caspase in adult brains. Survival to adulthood and adult lifespan are more severely impaired with decreasing larval age at the time of irradiation. The genetic background of the irradiated larvae affects survival to adulthood and adult motor ability, demonstrating that there is a genetic component to radiation sensitivity.

**Implications and future directions**

This work demonstrates the usefulness of *Drosophila* to model the toxic effects of radiation during development with the potential to unravel underlying mechanisms and to discover novel therapeutic interventions. Future screening of the *Drosophila* Genetic Reference Panel (DGRP; a library of *Drosophila* inbred lines that are fully sequenced) will allow for quantitative trait locus (QTL) mapping of genetic variants that alter radiosensitivity of survival and motor function. From this screen candidate genes can be identified and tested for their ability to alter these radiation-sensitive phenotypes. Once genes of interest are confirmed, the molecular pathways underlying radiation-induced neurotoxicity can be dissected to identify therapeutic targets for reducing damage to healthy tissue during radiation therapy.

## RESULTS

### Radiation during development reduces survival to adulthood

**Fig. 1.**
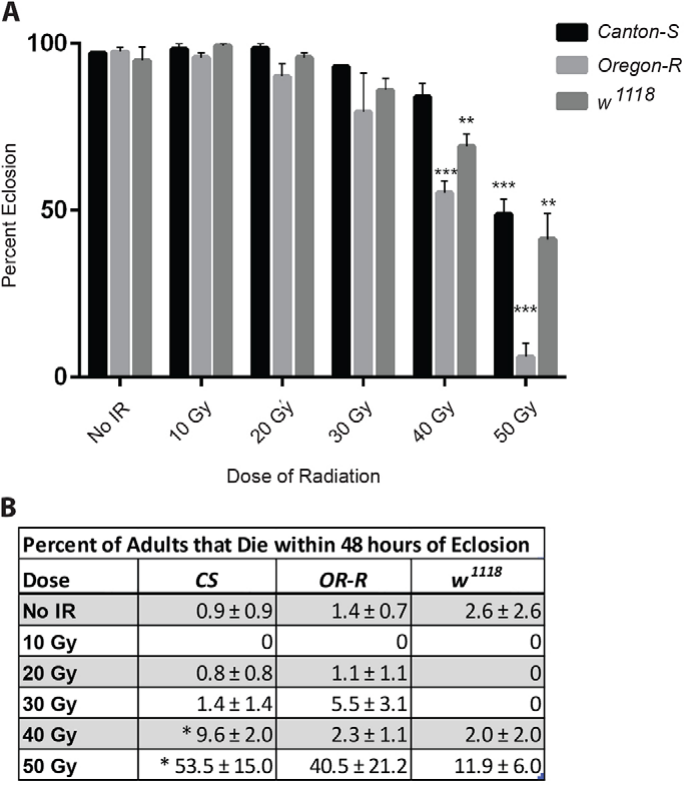
**Irradiation of larvae during the late third-instar reduces survival to adulthood.** (A) For each dose of radiation, the percentage of irradiated late third-instar larvae that completed development and eclosed as adults is shown for three different laboratory strains. Values shown are mean±s.e.m. for three independent trials of 30-50 larvae per dose per trial. *P<0.05, **P<0.01, ***P<0.001 based on Student's *t*-test comparing 40 Gy and 50 Gy to untreated (No IR). (B) The percentage of adult survivors from A that died within 48 h following eclosion. Values shown are mean±s.e.m. *P<0.05 based on Student's *t*-test comparing *Canton-S* treated with 40 Gy or 50 Gy with untreated (No IR).

*Drosophila* develop from embryos to adults in approximately 10 days at 25°C. Upon hatching, first-instar larvae feed, grow and undergo two successive molts to become third-instar larvae over the next two days. After another 2.5-3 days, metamorphosis begins with the onset of pupariation. Five days later, mature adults emerge from pupal cases. The majority of neurons in the adult central nervous system are derived from neural precursors originating in a post-embryonic wave of neurogenesis that peaks in mid-third-instar larvae (Ito and Hotta, 1992; Siegrist et al., 2010; Truman and Bate, 1988). To investigate how radiation exposure during development affects adult outcomes, we irradiated larvae in the late (wandering) third-instar stage. Varying doses of γ-radiation were delivered in a single fraction from a ^137^Cs source. Following irradiation, larvae were allowed to complete development, and the percentage of surviving adults was quantified. Consistent with previous studies (Balock et al., 1963), we found that doses of radiation up to 30 Gy had little effect on subsequent survival (Fig. 1A). At higher doses, survival to adulthood was reduced; exposure to 40-50 Gy reduced the percentage of adult survivors by 50%. Like other insects, *Drosophila* are remarkably resistant to radiation compared with mammals, but like mammals, they are more sensitive to radiation during development than in adulthood (Balock et al., 1963; Grahn, 1958; Ogaki and Nakashima-Tanaka, 1966; Parsons et al., 1969; Vaisnav et al., 2014; Westerman and Parsons, 1973).

### Radiation during development reduces adult lifespan

**Fig. 2.**
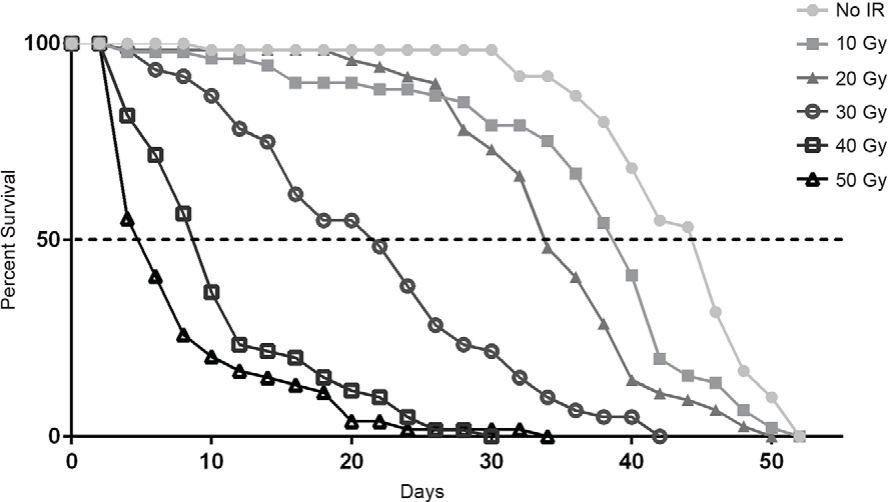
**Irradiation during larval development reduces the lifespan of surviving adults.** Percent survival at 29°C versus adult age (days after eclosion) is graphed for each dose of radiation administered during the late third-instar. Only flies surviving the first 48 h of adulthood were included in this analysis. Median lifespan is marked with the dashed line. Median survival was 5, 9, 21, 34, 39 and 44 days for 50 Gy, 40 Gy, 30 Gy, 20 Gy, 10 Gy and untreated (No IR), respectively. Each survival curve is based on three independent trials of 10-20 male *Canton-S* flies each. Data points on the graph represent mean percent survival. *P*-values from a log-rank test on each independent trial comparing untreated (No IR) versus 30 Gy were: 5.68×10^−11^, 1.9×10^−8^ and 7.8×10^−7^.

To determine whether radiation exposure during larval development has long-term effects on flies that are able to complete development, we measured the lifespan of surviving adults. At 50 Gy, the highest dose of radiation tested, we observed substantial adult mortality (>40% for *Canton-S* and *Oregon-R*) within 48 h of eclosion (Fig. 1B). Because this very early adult mortality is likely to be the result of residual developmental defects caused by radiation exposure, we excluded flies that died within 48 h of eclosion from subsequent lifespan measurement. Survival curves for the remaining *Canton-S* adults demonstrated that lifespan decreased as radiation dose increased (Fig. 2). These results also indicate that radiation during larval development has a greater effect (higher radiosensitivity) on the lifespan of surviving adults than it does on the survival of irradiated larvae to adulthood. For example, irradiation of *Canton-S* larvae at 30 Gy and 40 Gy reduces the median lifespan of the surviving adults by more than 50% compared with that of non-irradiated controls, even though these doses cause only a modest reduction on the percent of larvae that eclose (93% and 84% eclosion at doses of 30 Gy and 40 Gy, respectively).

### Radiation sensitivity increases with decreasing age of exposure

**Fig. 3.**
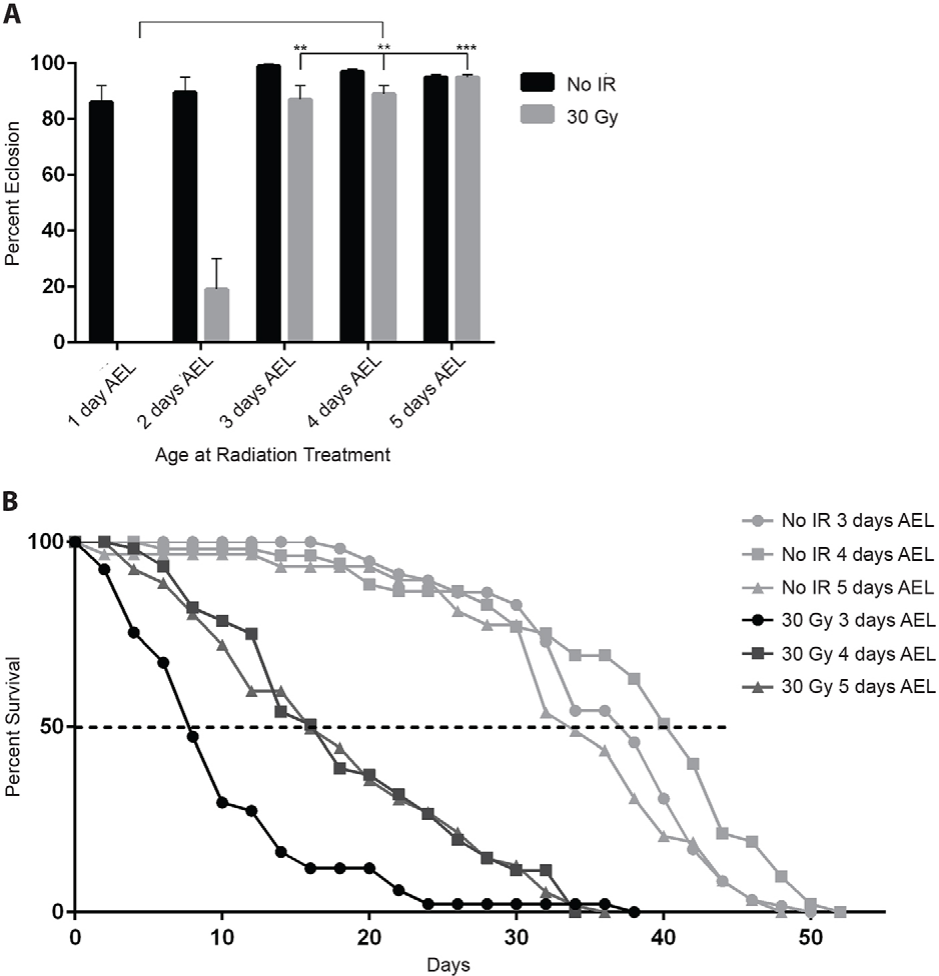
**Radiation sensitivity depends on the developmental stage.** (A) Percentage of larvae irradiated (30 Gy) at the indicated time points that completed development and eclosed as adults. Values shown are mean±s.e.m. for two independent trials, each comprising 30-50 *Canton-S* larvae. **P<0.01, ***P<0.001 based on Student's *t*-test comparing 3 days AEL, 4 days AEL or 5 days AEL to 1 day AEL (30 Gy). (B) Percent survival at 29°C versus the adult age (days after eclosion) is graphed for survivors of radiation (30 Gy) administered at the indicated time points, along with that of unirradiated controls. Only flies that survived the first 48 h of adulthood were included in this analysis. Median lifespan is marked with the dashed line. Median survival was 7, 16 and 16 days for 30 Gy 3 days AEL, 4 days AEL and 5 days AEL, respectively. Each survival curve is based on three independent trials of 10-20 male *Canton-S* flies each. Data points on the graph represent mean percent survival. *P*-values from log-rank tests on each independent trial comparing 30 Gy 3 days AEL and 30 Gy 5 days AEL were: 0.0187, 0.00412 and 0.00693.

In pediatric CRT, there is an inverse correlation between the age of the individual upon treatment and the severity of the long-term neurocognitive side effects from radiation (Edelstein et al., 2011b; Mulhern et al., 2005). To determine whether a similar relationship occurs in *Drosophila*, we examined the effect on adult eclosion of radiation exposure at different stages of larval development. We irradiated larvae at various developmental stages, from 1 to 5 days after egg lay (AEL) and quantified survival to adulthood, as well as the lifespan of survivors. We chose a dose of 30 Gy because it has little effect on survival to eclosion when larvae are irradiated at 5 days AEL but has strong effects on the lifespan of surviving adults (Figs 1 and 2). Consistent with the observations in humans, we found that as the age of larvae at the time of exposure decreased, the sensitivity to radiation increased (Fig. 3). For example, the fraction of larvae surviving to adulthood exceeded 85% when larvae were irradiated at 3-5 days AEL. By contrast, irradiation at 2 days AEL decreased adult survival to less than 20%, and there was a complete absence of survivors at 1 day AEL (Fig. 3A).

Although the percentage of adult survivors was similar when larvae between 3 and 5 days AEL were irradiated at 30 Gy, the lifespan of the surviving adults differed depending on the larval age at the time of exposure. In particular, adult survivors of larvae that had been irradiated at 3 days AEL had significantly shorter lifespans than adult survivors of larvae that had been irradiated at 4 or 5 days AEL (Fig. 3B). Once again, these results parallel results in humans following CRT with the side effects of radiation exposure becoming more severe as the time interval since treatment increases (Edelstein et al., 2011a).

### Radiation during development causes behavioral deficits in adults

**Fig. 4.**
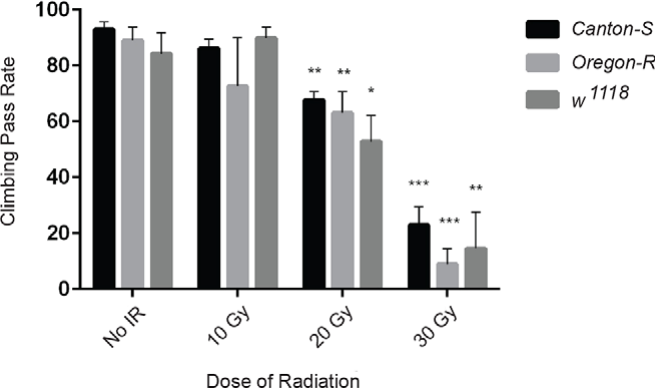
**Irradiation during larval development results in a dose-dependent decrease in locomotor ability in adults.** The climbing pass rate was assayed for adult males from three laboratory strains following irradiation of late third-instar larvae*.* Each group (*N*≤11) of 5- to 6-day-old adults was tested five times with 1-min recovery periods between tests. Three independent trials were performed for each strain at each dose. The histogram shows the percentage of flies that climbed above 5 cm in 10 s for each strain. Values shown are mean±s.e.m. *P<0.05, **P<0.01, ***P<0.001 based on Student's *t*-test comparing 20 Gy and 30 Gy to untreated (No IR).

A particularly unfortunate consequence of pediatric CRT is neurocognitive, motor and behavioral disorders later in life. To determine whether irradiation of *Drosophila* larvae during development also causes neurological deficits at a later stage in life, we assayed the locomotor ability of adult flies as a convenient behavioral readout (Fig. 4). Wild-type flies are negatively geotactic, and after gentle tapping so that the flies drop to the bottom of the culture vial, the flies will quickly climb towards the top. To quantify climbing ability, we determined the fraction of flies that climbed past a line marking a vertical distance of 5 cm from the bottom of the vial within 10 s. This assay provides a good assessment of overall neurological function because proper performance requires sensory input, central processing and motor output. We observed that larval irradiation resulted in behavioral impairment of adult survivors, with increasing impairment at higher doses (Fig. 4). Like adult lifespan, climbing behavior was a more radiosensitive phenotype than survival to adulthood following irradiation of larvae. Thus, although irradiating late third-instar larvae at 20 Gy had only a minimal effect on survival to adulthood, the climbing pass rate of surviving adults was reduced by more than 25% compared with non-irradiated controls. We did not determine a climbing pass rate for adults that survived larval irradiation at 40 or 50 Gy because their locomotor behavior was so severely impaired that these flies hardly climbed at all.

### Radiation during development causes cell death in adult brains

**Fig. 5.**
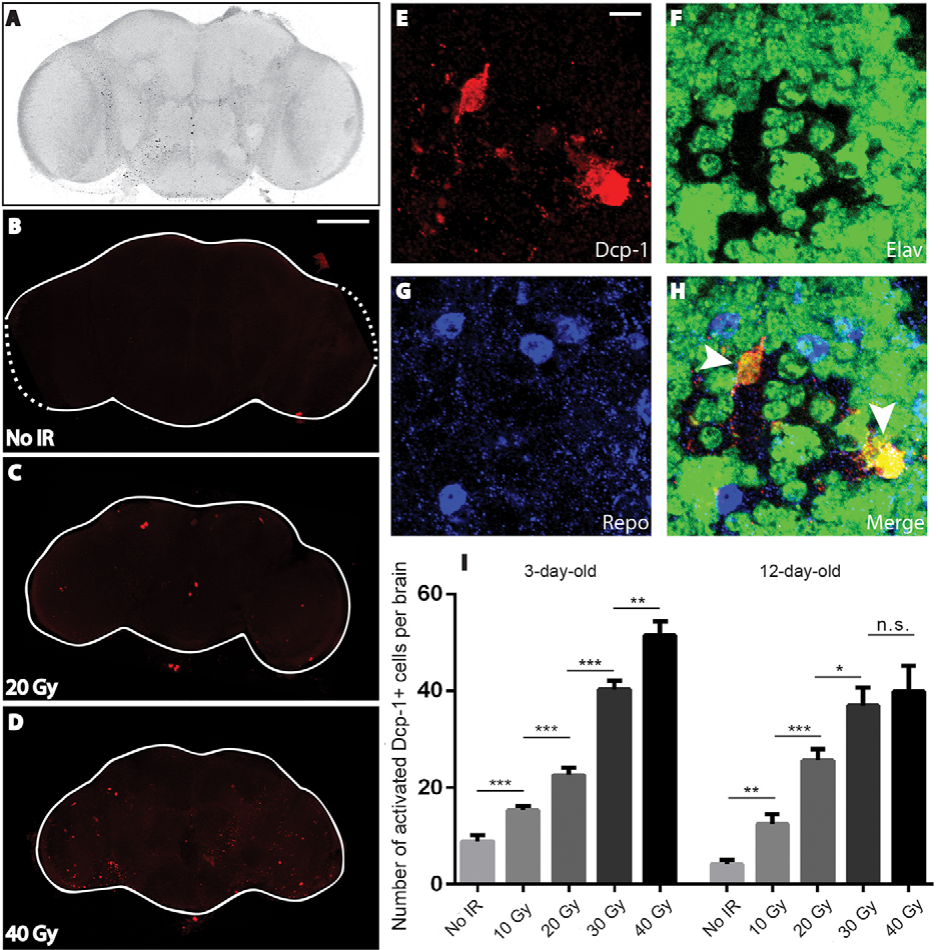
**Irradiation during larval development increases the frequency of cells expressing a cell death marker in adult brains.** Following exposure of late third-instar *Canton-S* larvae to the indicated doses of radiation, surviving adults were aged for 3 days after eclosion before brains were dissected for confocal microscopy. (A-D) Confocal stacks of whole brains immunostained for activated Dcp-1. (A) Grayscale image of a non-irradiated (No IR) brain with enhanced contrast to show anatomy. (B-D) Red-green-blue (RGB) images showing activated Dcp-1-positive cells (red). (E-H) Cells with activated Dcp-1 in adult brains following larval irradiation were predominantly neurons not glia. Higher magnification confocal stacks of 3-day-old adult *Canton-S* brains following irradiation of late third-instar larvae at 40 Gy stained for activated Dcp-1 (E), the neuronal nuclear marker Elav (F) and the glial nuclear marker Repo (G). Dcp-1 colocalized with Elav but not Repo (arrowheads) (H). (I) Quantification of the number of Dcp-1-positive brain cells in 3-day-old and 12-day-old adult *Canton-S* flies following irradiation of third-instar larvae at the indicated dose. Two independent assays of 5-8 brains per dose were performed. Values shown are mean±s.e.m. *P<0.05, **P<0.01, ***P<0.001 based on Student's *t*-test comparing the number of activated Dcp-1-positive cells at a given dose of radiation with the corresponding number at the previous lower dose. ns, not significant. Scale bars: 100 µm (B-D), 5 µm (E-H).

Our studies on lifespan and behavior of adult flies following larval irradiation demonstrate that in *Drosophila*, as in humans, radiation exposure can have persistent, long-term effects resulting in neurological impairment as well as reduced lifespan. We hypothesized that at least some of the damaging effects of radiation on the nervous system would be observable at the cellular level. To test this hypothesis, we irradiated late third-instar larvae, dissected brains from the resultant adults after aging them for three days and stained them for activated Death caspase-1 (Dcp-1), a *Drosophila* effector caspase (Hay and Guo, 2006), which along with Death-related ICE-like caspase (Drice) is a commonly used marker for cells undergoing apoptosis in *Drosophila* (Hay and Guo, 2006; Florentin and Arama, 2012). We observed a dose-dependent increase in the number of activated Dcp-1-labeled cells compared with non-irradiated controls, indicating that there was an increase in the number of cells undergoing apoptosis in adult brains following larval irradiation (Fig. 5). We observed a similar dose-dependent increase in Dcp-1-positive cells in the brains of 12-day-old adults (e.g. approximately 2.5 weeks after irradiation) (Fig. 5). Although there were fewer Dcp-1-labeled cells in the brains of 12-day-old flies compared with 3-day-old flies, their level was still elevated compared with controls, demonstrating that an early exposure to radiation has ongoing deleterious consequences much later in life. Co-labeling cells with antibodies against Repo and Elav to mark glia and neurons, respectively (Halter et al., 1995; O'Neill et al., 1994), revealed that most of this Dcp-1 activation occurred in neurons (Fig. 5E-H). The cumulative effect of continuous apoptosis in adult brains over several weeks is very likely to be a major factor in the neurological impairment we observe in these flies following larval irradiation, which might become more severe with time since irradiation.

### Sensitivity to radiation depends on genetic background

An important goal in developing a *Drosophila* model of radiation-induced neurotoxicity is to identify and unravel the key molecular mechanisms that underlie the observed effects of radiation on viability and neurological function. In particular, because of the genetic tools available in *Drosophila*, we can easily ask whether there are genetic differences in sensitivity to the effects of radiation and, if so, identify variants in specific genes that confer radioresistance or radiosensitivity to discover the proteins and pathways that play major roles in mediating the observed effects of radiation. One way to discover such variants is by conducting a screen following chemical mutagenesis for new mutants that are more sensitive or resistant to radiation than wild-type flies. Another approach is to take advantage of existing variation in natural populations to screen for the phenotypes of interest. This latter approach is facilitated by the *Drosophila* Genetic Reference Panel (DGRP), a collection of over 200 sequenced highly inbred lines, each generated from single females captured from a natural population (Huang et al., 2014; Mackay et al., 2012). Thus, each line represents the genetic variants present in the genome of a single fly. Because the genome for each line has been fully sequenced, these lines are an extremely valuable resource for association mapping, in which a phenotype of interest can be correlated with variants in a specific gene. Investigators have previously used these lines to identify candidate genes involved in a variety of biological mechanisms, including sleep, longevity and olfactory behavior (Durham et al., 2014; Harbison et al., 2013; Swarup et al., 2013).

**Fig. 6.**
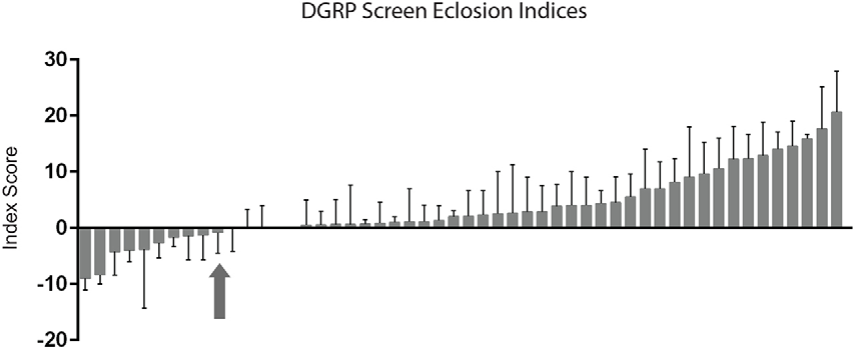
**Genetic background affects radiosensitivity.** Bar graph showing the eclosion index for each of the 52 DGRP lines that were assayed for sensitivity to radiation, organized from the lowest to the highest index. Values shown are mean±s.e.m. for two to four independent trials of 20-30 larvae each. *P*=0.0056 based on a one-way ANOVA. Arrow indicates where the *Canton-S* eclosion index falls.

We performed an initial survey of the DGRP lines to determine whether this collection harbors genetic variants that affect survival to eclosion following larval irradiation. For this analysis we determined an ‘eclosion index’ (see Materials and Methods) for 52 DGRP lines following a 20 Gy dose of radiation during the late third-instar. We observed significant variation in the effect of radiation on survival to eclosion among the DGRP lines tested (Fig. 6). To expand our analysis of the effect of genetic background on radiation sensitivity, we also examined a subset of the DGRP collection for the climbing behavior of adults after irradiating late third-instar larvae at 20 Gy. Single-trial data suggested that the effect of radiation on the climbing behavior of adults, measured by calculating a ‘climbing index’ (see Materials and Methods), also varied over a wide range among the DGRP lines tested (data not shown). An unexpected finding was that the eclosion index or climbing index for some DGRP lines had a negative value, indicating that survival to adulthood or locomotor activity improved following irradiation compared with that of unirradiated controls. At this point, we remain uncertain about the significance of this result. Although there are plausible biological mechanisms by which moderate doses of radiation could confer some benefit to particular genetic variants, we cannot rule out the possibility of experimental artifact. In addition, in at least several instances, negative indices were associated with DGRP lines whose survival to eclosion or locomotor ability was poor, even without irradiation. The observed results could thus be more indicative of a poor starting phenotype than real benefits of irradiation. Further experiments will be necessary to investigate these possibilities. Examination of the eclosion index results shown in Fig. 6 and of our preliminary climbing index data (not shown) also indicates that, although some lines appear to be more resistant to radiation than our standard wild-type strain both for eclosion and climbing ability, overall there does not appear to be a strong correlation between these two indices in the various lines tested. These results suggest that although variants in some genes might confer overall radiation resistance, it is more common for radiosensitivity of eclosion to be affected by a different set of genetic variants from those affecting radiosensitivity of locomotor activity. Thus, it is likely that the deleterious effects of radiation on these two phenotypes are mediated by distinct cellular mechanisms.

Overall, these results demonstrate that survival to adulthood following larval irradiation is a quantitative trait that is dependent on genetic background. Moreover, genetic variants affecting this trait appear to be well represented in the DGRP lines, suggesting that additional screening will be useful for identifying specific genes that affect the long-lasting damage induced by radiation exposure during development.

## DISCUSSION

The goal of this work was to establish a *Drosophila* model for investigating the neurotoxic side effects of radiation therapy during development. Exposing late third-instar larvae to radiation results in a dose-dependent decrease in survival to eclosion, shortened adult lifespan, impaired locomotion and increased cell death in the adult brain. The effects of radiation on eclosion and adult lifespan are more severe when larvae are irradiated at progressively earlier stages in development. These findings parallel those in humans, where neurological side effects worsen with increasing radiation dose (Redmond et al., 2013) and the lower the age at the time of radiation (Edelstein et al., 2011b; Mulhern et al., 2005). Thus, many of the most important deleterious effects of CRT in humans are paralleled in *Drosophila*, suggesting that the *Drosophila* model can be useful in elucidating key genetic mechanisms responsible for the neurotoxic effects of radiation that are likely to be conserved between flies and humans.

Despite the experimental advantages of using *Drosophila* as an experimental model, it is important to recognize that there are significant biological and technical differences between our radiation-exposure model in flies and CRT in humans that must be considered when trying to extrapolate from flies to humans. One important difference is that unlike CRT, where radiation is targeted to particular regions of the brain, we can only expose entire larvae to irradiation because of their small size. Thus, although we are particularly interested in the deleterious effects of radiation on the nervous system and focus on phenotypes such as climbing behavior and neuronal cell death in the brain, we cannot be certain that these phenotypes and others, such as successful eclosion, do not involve effects of irradiation on other tissues. Nonetheless, all the phenotypes we focus on are known to have a substantial dependence on the nervous system, and it is a reasonable working hypothesis to assume that these phenotypes are at least providing some measure of neural function following radiation exposure. Ultimately, by identifying specific genetic variants that confer different radiosensitive phenotypes, we can test the validity of our hypothesis by manipulating expression of these genes in a tissue-specific manner.

It has been proposed that many of the neurological side effects of CRT are due to a decline in neurogenesis following radiation (Monje et al., 2002; Kempf et al., 2014; Son et al., 2015). Although there is considerable neurogenesis in the adult mammalian brain (Aimone et al., 2014), the adult fly brain is largely, although not entirely, post-mitotic (Siegrist et al., 2010; von Trotha et al., 2009). Consequently, it would be difficult to detect a decrease in neurogenesis in the *Drosophila* brain following radiation exposure. By contrast, in the transition from the larval nervous system to the adult nervous system that takes place during pupariation and metamorphosis in *Drosophila*, there is continued neurogenesis for about three days (Siegrist et al., 2010), which could be impacted by radiation exposure during the larval stage. Thus, although a *Drosophila* model might not be able to precisely replicate every feature of CRT in humans, this is also true in the majority of instances where *Drosophila* has been used to model human biological and disease mechanisms, including embryonic pattern formation, sleep, circadian rhythm, cancer, epilepsy, retinal degeneration, etc. (Nusslein-Volhard and Wieschaus, 1980; Potdar and Sheeba, 2013; Hardin, 2011; Rudrapatna et al., 2012; Cunliffe et al., 2015; Xiong and Bellen, 2013). With awareness of the limits of the model and appropriate caution in data interpretation, a *Drosophila* model, although imperfect, can nonetheless provide powerful new insights and starting points for unraveling normal and pathological biological mechanisms in humans.

Another important difference in our experimental model is that we delivered radiation exposure in a single, unfractionated dose to late third-instar larvae, whereas CRT generally utilizes fractionated exposure delivered in smaller doses over many days to achieve a final total dose to reduce toxicity to normal tissue while effectively killing malignant cells (Ahmed et al., 2014). Because the third-instar in *Drosophila* larvae only lasts for 2-3 days, there is a very narrow time window over which radiation exposure could be fractionated. Moreover, because *Drosophila* are inherently more radioresistant than humans and can readily tolerate much higher doses of radiation, there is no particular reason to fractionate the doses of radiation used in our experiments. Whether this difference in radiation delivery between our fly model and CRT is of sufficient mechanistic significance that there would be no overlap between the genes and signaling pathways in flies and humans that confer radioresistance remains to be determined. Nonetheless, it seems reasonable to assume that fundamental biological consequences of radiation on the nervous system will be conserved between flies and humans regardless of fractionation. Therefore, we believe it is likely that identification of radioresistant genes in *Drosophila* will ultimately still be valuable in unraveling key pathways that are responsible for long-term sequelae of CRT in humans.

One important area where a *Drosophila* model of CRT can play a particularly important role is in the discovery and development new radioprotective agents that have potential for clinical application. Although radioprotective thiols have been studied for decades, their clinical application has been limited owing to their toxic side effects and their ineffectiveness when administered orally (Hosseinimehr, 2007). One of the most important strengths of a *Drosophila* model is that it can be used for unbiased genetic screens to identify key molecular players underlying radiation-induced damage. Our initial screen of the DGRP lines indicates that *Drosophila* can be used to identify genetic variants that confer large differences in radiation sensitivity. Because of the genetic tools available in *Drosophila*, once candidate genes for these differences in radiosensitivity are identified, the relevant molecular pathways can be readily studied. Understanding these pathways and their molecular components provides a rational strategy for identification of novel therapeutic targets. Moreover, with the growing emphasis on genome-sequence-driven personalized medicine, knowledge of which genetic variants confer greater or lesser sensitivity to the deleterious side effects of radiation should enable the development of CRT regimens that are specifically tailored to individuals.

In addition to the expected immediate deleterious effects of CRT, a much more puzzling consequence is the ongoing long-term effects, which are still evident years or decades after treatment. For example, magnetic resonance spectroscopic analysis of children that have undergone CRT has demonstrated metabolic changes that are associated with brain tissue damage up to 18 months after completion of therapy (Blamek et al., 2010). Magnetic resonance diffusion tensor imaging of adults 25 years after CRT has revealed loss of white matter integrity compared with healthy controls (Schuitema et al., 2013). The cellular targets and the cascade of events triggered by irradiation that are responsible for these persistent long-term toxic effects remain very poorly understood. It is therefore of interest that we observe comparable long-term effects in our *Drosophila* model. Notably, when we examined adult brains following irradiation of third-instar larvae, we found a dose-dependent elevation of activated-caspase staining compared with unirradiated controls, not only in 3-day-old adults but also in 12-day-old adults, e.g. 17 days after irradiation, suggesting an increase in cell death. With a mean lifespan for control flies in our experiments of approximately 45 days, a timespan of 17 days would be roughly equivalent to decades in humans. Therefore, one might have expected that cell death would be sharply elevated immediately after irradiation and that the process would be completed a short time thereafter. Although the number of cells with activated Dcp-1 was markedly lower in the brains of 12-day-old adults compared with 3-day-old adults following larval irradiation, it was still significantly higher than that in unirradiated controls. These results strongly suggest that larval irradiation not only has immediate effects resulting in cell death, but also triggers some kind of persistent dyshomeostasis in cells that survive the acute effects of irradiation resulting in their eventual death at a much later time.

The long-term neurotoxic side effects of CRT are one of the most difficult issues to confront in treating children with cancer. However, if we can gain a better understanding of the cellular and molecular basis of these delayed effects, it might open a large window for therapeutic intervention to reduce the side effects of CRT in children. For example, our observation that brain cells continue to undergo programmed cell death in adult flies 17 days after irradiation suggests that, after the desired tumor-killing benefits of CRT have been achieved in children, there might be an opportunity to initiate appropriate therapy that would limit subsequent ongoing damage to healthy cells and thereby mitigate toxic side effects. To achieve such a favorable outcome, it will be necessary to have a much more detailed understanding of the persistent toxic cascade triggered by irradiation, and the genes and protein products responsible for orchestrating this long-lasting toxicity. As in the search for new radioprotective compounds, we believe that the genetic and molecular tools available in *Drosophila* offer a powerful and systematic approach that can help provide the missing information.

## MATERIALS AND METHODS

### *Drosophila* strains and culture

Flies were maintained on standard cornmeal-molasses medium at 25°C, except for lifespan assays, which were performed at 29°C. Two standard wild-type lines (*Canton-S* and *Oregon-R*) and one common white-eyed background strain (*w^1118^*) were used as controls. The DGRP (Mackay et al., 2012) was used for experiments in Fig. 6. For all experiments involving irradiation of late third-instar larvae, adults were allowed to mate and lay eggs in culture bottles on standard medium, and then cleared from the bottles after 4-5 days to prevent larval crowding. Wandering third-instar larvae were collected for irradiation when they emerged from the medium. Known numbers of larvae were placed in culture vials with an absorbent tissue at the bottom to reduce excess moisture and to prevent newly eclosed flies from getting stuck.

### Irradiation of late third-instar larvae

Vials containing 30-50 late third-instar larvae were placed on a rotating plate and exposed to a single dose of γ-radiation (10, 20, 30, 40 or 50 Gy) using a ^137^Cs irradiator (J. L. Sheppard and Associates, San Francisco, CA; Mark I unit, Model 30, Serial Number 668) with an average dose rate of 6.5 Gy/min. Control larvae were handled identically but without radiation exposure. Irradiated and control larvae were then allowed to complete development at 25°C, and all adults that subsequently emerged were collected and counted.

### Irradiation of larvae at earlier developmental stages

Cultures of *Canton-S* adults were allowed to lay eggs on small Petri dishes containing apple-juice-agar medium. Approximately 24 h later (1 day AEL), newly hatched first-instar larvae were collected and transferred to fresh apple juice plates covered in standard cornmeal-molasses medium, and allowed to develop at 25°C until the specified time of irradiation. For irradiation of first-instar larvae, they were transferred directly after hatching to fresh vials containing standard medium. For irradiation of larvae at later stages, they were collected, counted and transferred to vials containing approximately 6 ml of standard medium, softened by the addition of 1-2 ml of water. In all cases, control vials were handled identically but without irradiation.

### Lifespan analysis

Newly eclosed adults were collected, sorted by sex and transferred to fresh vials (10 males or females per vial) containing standard medium and aged at 29°C. Flies were transferred to fresh food every other day, at which time the number of surviving flies was recorded. Adult flies that died within 48 h of eclosion were counted but excluded from lifespan analysis. Males and females both demonstrated dose-dependent radiation sensitivity in all of our assays. However, as many studies on lifespan in *Drosophila* show, there are a number of biological differences between males and females that affect survival for reasons unrelated to the investigation at hand. In particular, because so much of the physiology of females is dedicated to egg production, we wanted to eliminate this source of variability and, for the studies presented here, concentrated primarily on analyzing males (Bonduriansky et al., 2008).

### Climbing assay

Newly-eclosed adults were collected and aged in vials of standard medium at 25°C (≤11 flies of the same sex per vial) for 5-6 days before testing. For the behavioral test, flies (*N*≤11) of the same sex were transferred to the climbing apparatus, which comprised two empty plastic culture vials taped together at the open end (vial diameter: 2.3 cm, height: 9.3 cm). After a 1-min recovery, flies were gently tapped to the bottom, and the number that climbed vertically beyond a 5-cm mark within 10 s was recorded. The test was repeated five times for each cohort of flies with 1-min rest periods between each test (adapted from Ali et al., 2011).

### Immunohistochemistry

Adult brains were dissected in 1× PBS and fixed at room temperature for 25-30 min in 4% formaldehyde in phosphate buffer [4% formaldehyde, 0.1 M phosphate buffer (pH 7.2), 0.2% Triton X-100]. Samples were then washed with 1× PBS and placed in blocking buffer (PBS, 0.2% Triton X-100, 0.1% normal goat serum) for 2 h at room temperature or 4°C overnight. Brains were incubated with primary antibodies diluted with blocking buffer at 4°C overnight, then washed twice with PBST (PBS+0.1% Triton X-100) and incubated in secondary antibodies diluted with blocking buffer for 4 h at room temperature, then washed twice again with PBST. DAPI was added in the final wash for 30 min at room temperature. Brains were mounted on slides in Vectashield (Vector Laboratories, Burlingame, CA). The antibodies used are as follows: rabbit anti-cleaved Dcp-1 (Cell Signaling Technology, Danvers, MA; #9578, 1:100), mouse anti-Repo (Developmental Studies Hybridoma Bank, University of Iowa, Iowa City, IA; 8D12, 1:50), Rat anti-Elav (Developmental Studies Hybridoma Bank, University of Iowa, Iowa City, IA; 7E8A10, 1:250), DAPI (Sigma-Aldrich, St Louis, MO; 1 mg/l), goat anti-mouse Alexa-Fluor-488 (Life Technologies, Carlsbad, CA; 1:200), goat anti-rabbit Alexa-Fluor-568 (Life Technologies, Carlsbad, CA; 1:200), goat anti-rat Alexa-Fluor-633 (Invitrogen, Carlsbad, CA; 1:200).

### Microscopy

Adult brains were imaged using a Zeiss 510 Confocal Laser Scanning Microscope (Carl Zeiss Microscopy, Jena, Thuringia, Germany). ImageJ Software (National Institutes of Health, Bethesda, MD) and Adobe Photoshop CC (Adobe Systems, San Jose, CA) were used for adjusting image brightness and contrast.

### Calculation of indices for DGRP lines

Eclosion index=percent eclosion [non-irradiated]−percent eclosion [20 Gy]. Climbing index=climbing pass rate [non-irradiated]−climbing pass rate [20 Gy].

### Statistical analyses

GraphPad Prism (GraphPad Software, San Diego, CA) was used for Student's *t*-test analyses of eclosion, climbing and Dcp-1-positive cells, and for one-way ANOVA of the DGRP eclosion indices. The DLife software package (Scott Pletcher University of Michigan, Ann Arbor, MI) was used for log-rank tests on survival curves.
